# A243 THROMBOELASTOGRAPHY-GUIDED COAGULOPATHY CORRECTION IN CIRRHOTIC PATIENTS DECREASES BLOOD PRODUCT TRANSFUSION: A SYSTEMATIC REVIEW AND ANALYSIS

**DOI:** 10.1093/jcag/gwac036.243

**Published:** 2023-03-07

**Authors:** H Komeylian, C Ching, J Zhu

**Affiliations:** 1 Digestive Health and Endoscopy, Dalhousie University, Halifax; 2 Faculty of Medicine, Dalhousie University, Dalhousie, Canada

## Abstract

**Background:**

Patients with cirrhosis are a risk factor for coagulopathy and bleeding complications. Thromboelastography (TEG) guided therapy is available to rapidly assess and guide blood product transfusion in this population.

**Purpose:**

This study aims to determine if TEG-guided therapy is able to decrease the administration of blood products and reduce adverse events such as bleeding and mortality compared to standard coagulation testing (SCT).

**Method:**

We performed a systematic review and meta-analysis of literature in PubMed, EMBASE, and The Cochrane Library and pooled six randomized-controlled trials comparing thromboelastography versus standard coagulation testing in patients with cirrhosis. A total of six studies were pooled (n= 386). Primary outcomes were mean platelet transfusion units and fresh frozen plasma (FFP) transfusion units. Secondary outcomes were mortality and bleeding events.

**Result(s):**

Compared to SCT, there was a significant standard mean decrease in platelet (P<0.00001) and FFP (P<0.00001) administration compared to TEG perioperatively prior to a liver transplant. However, there was no significant difference in the pooled odds ratio between TEG and SCT in patients receiving both platelets and FFP (OR 1.40 [95% Cl 0.61-3.23, P=0.42]). Recurrent variceal bleeding was significantly reduced in the TEG group compared to SCT for platelet transfusion (OR 0.05 [95% Cl 0.01-0.20, P<0.0001]) or FFP transfusions (OR 0.18 [95% Cl 0.05-0.63, P=0.007]). With regards to the 5-day, 28-day, 42-day, and 90-day mortality, there was no significant difference between the pooled odds ratio between the TEG and SCT groups (OR 0.69 [95% Cl 0.47-1.02, P=0.06]). The 24-hour, 48-hour, 5-day, and 42-day rebleeding rates were significantly lower in TEG versus SCT groups (OR 0.50 [95% Cl 0.29-0.85, P = 0.01)].

**Conclusion(s):**

The use of TEG-guided therapy favors a reduction in platelet and FFP transfusion compared to SCT in patients with cirrhosis. Additionally, TEG-guided therapy is able to improve patient care by reducing the rebleeding rate of patients with cirrhosis compared to SCT. However, this same effect was not seen for mortality.

**Please acknowledge all funding agencies by checking the applicable boxes below:**

None

**Disclosure of Interest:**

None Declared

